# Supplementary material for: Laser-Induced Graphene on Biocompatible PDMS/PEG Composites for Limb Motion Sensing
Source: Sensors (Basel). 2025 Aug 22;25(17):5238. doi: 10.3390/s25175238 (PMC12431460; doi:10.3390/s25175238)
Supplement: Supplementary file 1 [file sensors-25-05238-s001.zip › sensors-3746435-supplementary/sensors-3746435-supplementary.pdf]

Supplementary Materials

Laser-Induced Graphene on Biocompatible PDMS/PEG Composites for Limb Motion Sensing

Anđela Gavran[1]*, Marija V. Pergal[1], Teodora Vićentić[1], Milena Rašljić Rafajilović[1], Igor A. Pašti[2], Marko V. Bošković[1], and Marko Spasenović[1]*

[1] Center for Microelectronic Technologies, Institute of Chemistry, Technology and Metallurgy, National Institute of the Republic of Serbia, University of Belgrade, Belgrade, Serbia

[2] Faculty of Physical Chemistry, University of Belgrade, Belgrade, Serbia

Corresponding authors: M. Spasenović, A. Gavran, e-mail: marko.spasenovic@ihtm.bg.ac.rs, andjela.gavran@ihtm.bg.ac.rs

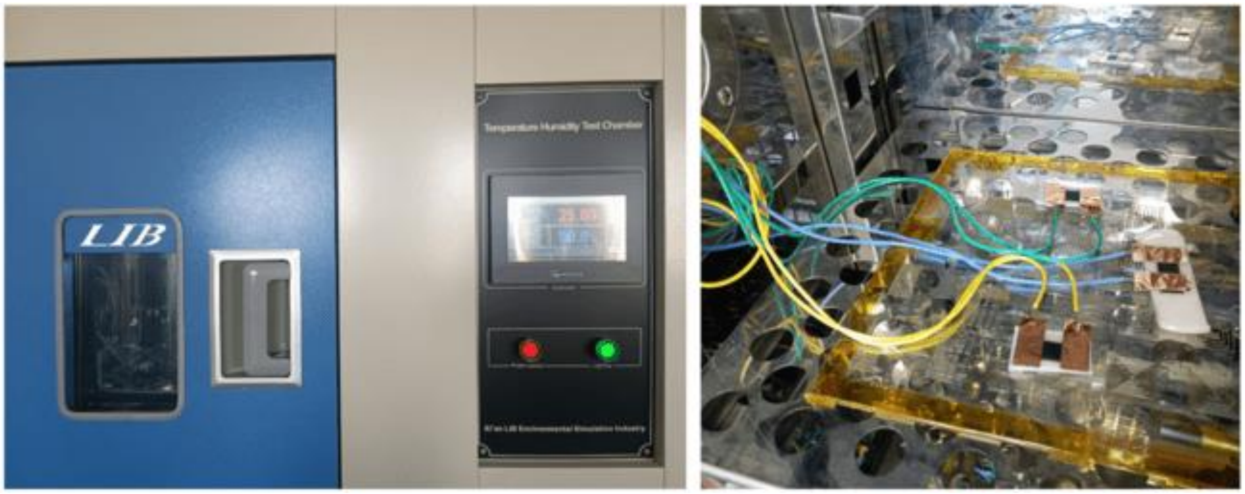

Figure S1. Photographs of humidity and temperature test chamber (left) and the tested samples in it (right)

Table S1. Tested laser parameters on PDMS/PEG for different PEG content

Sample Code[a]	PEG Content (wt. %)	Laser Power (W)	Laser Speed (mm s^{-1})	Resolution (DPI)	Line spacing (mm)	Laser Repetition (passes)
**LIG-10-15-35**	10	15	35	1200	9	1
**LIG-10-15-45**	10	15	45	1200	9	1
**LIG-10-15-55**	10	15	55	1200	9	1
**LIG-10-16-35**	10	16	35	1200	9	1
**LIG-10-16-45**	10	16	45	1200	9	1
**LIG-10-16-55**	10	16	55	1200	9	1
**LIG-20-15-35**	20	15	35	1200	9	1
**LIG-20-15-45**	20	15	45	1200	9	1
**LIG-20-15-55**	20	15	55	1200	9	1
**LIG-20-16-35**	20	16	35	1200	9	1
**LIG-20-16-45**	20	16	45	1200	9	1

**LIG-20-16-55**	20	16	55	1200	9	1
**LIG-30-15-35**	30	15	35	1200	9	1
**LIG-30-15-45**	30	15	45	1200	9	1
**LIG-30-15-55**	30	15	55	1200	9	1
**LIG-30-16-35**	30	16	35	1200	9	1
**LIG-30-16-45**	30	16	45	1200	9	1
**LIG-30-16-55**	30	16	55	1200	9	1
**LIG-40-15-35**	40	15	35	1200	9	1
**LIG-40-15-45**	40	15	45	1200	9	1
**LIG-40-15-55**	40	15	55	1200	9	1
**LIG-40-16-35**	40	16	35	1200	9	1
**LIG-40-16-45**	40	16	45	1200	9	1
**LIG-40-16-55**	40	16	55	1200	9	1

[a] – first number represents PEG content, second laser power, and third laser speed

Table S2. Electrical resistance of LIG/PDMS/PEG for different laser powers, fixed laser speed 45 mm s^{-1} and resolution 1200 DPI

	Electrical resistance (kOhm)			
**Laser power (W)**	LIG/PDMS/10%PEG	LIG/PDMS/20%PEG	LIG/PDMS/30%PEG	LIG/PDMS/40%PEG
**9**	125	108	24	0.5
**9.6**	112	100	11	0.9
**10.2**	247	215	26.7	31
**10.6**	6200	357	1920	250

Table S3. Electrical resistance of LIG/PDMS/PEG for different laser speed, fixed laser power 9 W and resolution 1200 DPI

	Electrical resistance (kOhm)			
**Laser speed (mm s^{-1})**	LIG/PDMS/10% PEG	LIG/PDMS/20% PEG	LIG/PDMS/30% PEG	LIG/PDMS/40% PEG
**35**	53	22	24	16.5
**45**	125	108	24	0.5
**55**	22	120	35	121

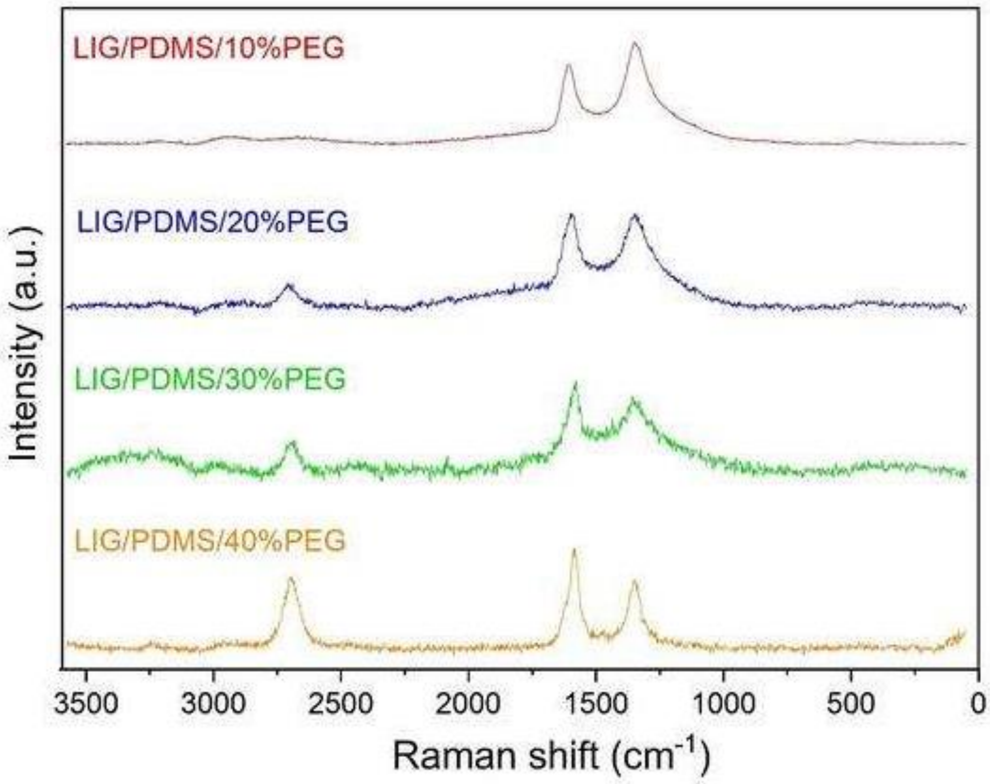

Figure S2. Raman spectra of LIG on PDMS with varying PEG content (10-40 wt.%) made with a scanning speed of 55 mm s^{-1}.

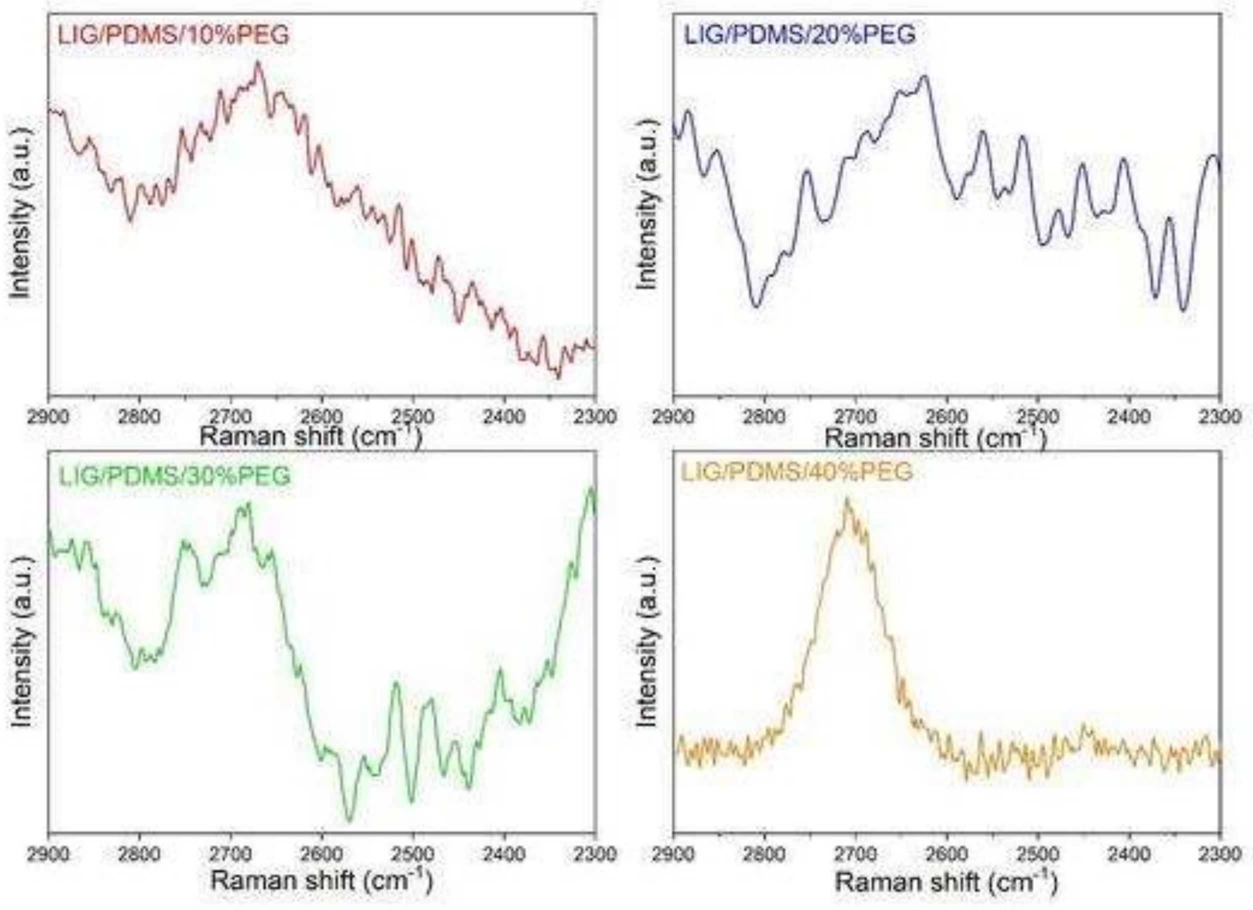

Figure S3. Raman spectra in the vicinity of 2D bands for LIG on PDMS with 10-40 wt.% PEG content, made with a scanning speed of 45 mm s^{-1}

Table S4. Positions of D, G, and 2D bands in Raman spectra of LIG/PDMS/PEG with different PEG content for LIG made with a scanning speed of 55 mm s^{-1}.

Materials	D band (cm^{-1})	G band (cm^{-1})	2D band (cm^{-1})
LIG/PDMS/10%PEG	1350.7	1603.6	2669.2
LIG/PDMS/20%PEG	1349.2	1587.5	2690.0
LIG/PDMS/30%PEG	1349.7	1587.9	2688.4
LIG/PDMS/40%PEG	1344.2	1581.9	2693.1

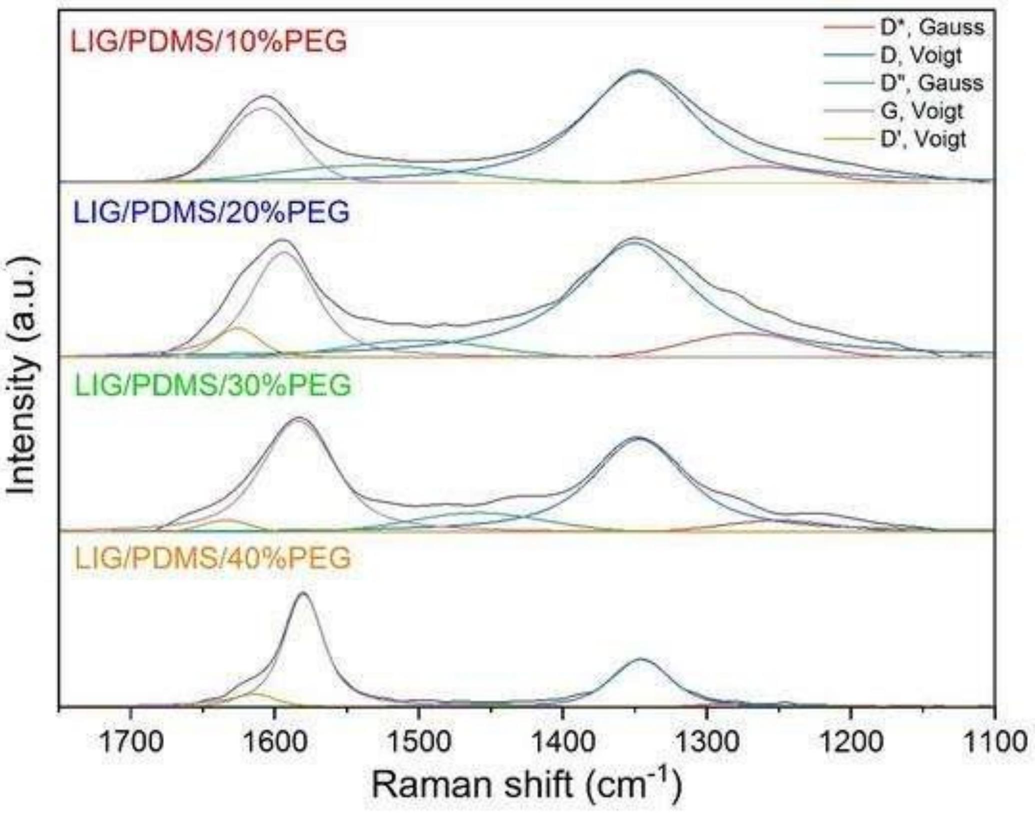

Figure S4. Deconvolution of five functions (D*, D, D'', G and D' bands) for LIG on PDMS with varying PEG content made with a scanning speed of 55 mm s^{-1}.

Table S5. I_D/I_G, I_{2D}/I_G, FWHM, and L_a obtained from Raman spectra of LIG/PDMS/PEG materials made with a scanning speed of 55 mm s^{-1}.

Materials	I_D/I_G	I_{2D}/I_G	FWHM (cm^{-1})		L_a (nm)
			D	G	
LIG/PDMS/10%PEG	2.9	0.001	83.5	57	6.6
LIG/PDMS/20%PEG	1.8	0.019	89	51.6	10.4
LIG/PDMS/30%PEG	1.2	0.026	83.3	63.2	15.5
LIG/PDMS/40%PEG	1.1	1.439	50.1	44.5	17.5

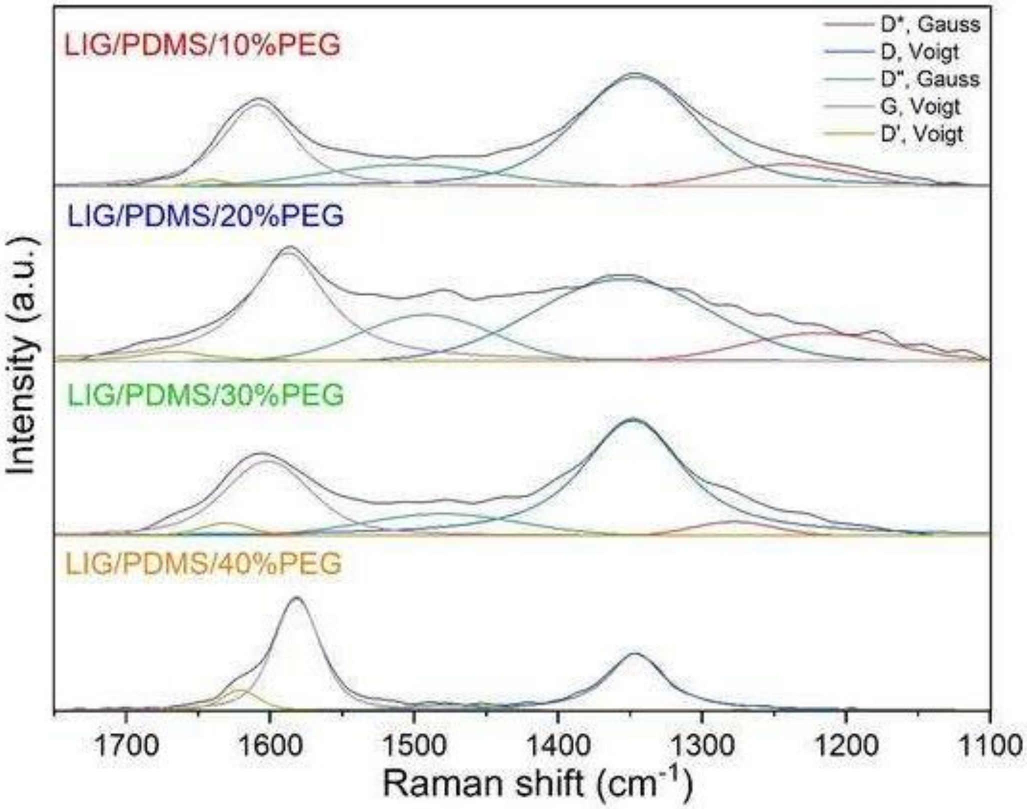

Figure S5. Deconvolution of five functions (D*, D, D'', G and D' bands) for LIG on PDMS with varying PEG content made with a scanning speed of 45 mm s^{-1}.

Table S6. Positions of D, G and 2D bands in Raman spectra of LIG/PDMS/PEG with different PEG content for LIG made with a scanning speed of 45 mm s^{-1}.

Materials	D band (cm^{-1})	G band (cm^{-1})	2D band (cm^{-1})
LIG/PDMS/10%PEG	1350.7	1603.6	2669.2
LIG/PDMS/20%PEG	1349.2	1587.5	2690
LIG/PDMS/30%PEG	1349.7	1587.9	2688.4
LIG/PDMS/40%PEG	1344.2	1581.9	2693.1

Table S7. Intensity ratio of D and G, and 2D and G band for LIG on PDMS/PEG made with a scanning speed of 45 mm s^{-1}.

Materials	I_D/I_G	I_{2D}/I_G	FWHM (cm^{-1})		L_a (nm)
			D	G	
LIG/PDMS/10%PEG	2.7	0.1	90	57.8	7
LIG/PDMS/20%PEG	2.3	0.4	124.8	67.4	8.2
LIG/PDMS/30%PEG	2.2	0.5	88.5	66	8.4
LIG/PDMS/40%PEG	1.1	0.9	67.2	51.5	17.6

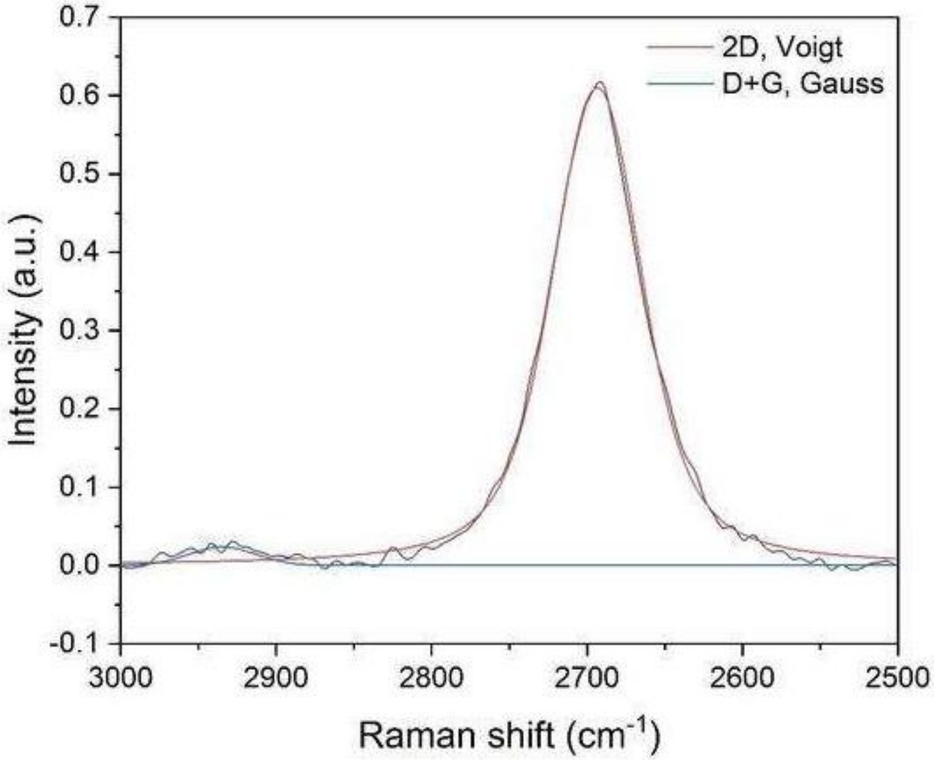

Figure S6. Deconvolution of the 2D and D+G regions for the Raman spectrum of LIG/PDMS/40%PEG.

Table S8. Assignation of bands in FTIR spectra of pure PDMS and LIG on PDMS/PEG with varying PEG content (10-40 wt.%).

Samples	*v* (O-H) [cm^{-1}]	*v* $(CH)_{as}$ [cm^{-1}]	*v* $(CH)_s$ [cm^{-1}]	*v* (C=C) [cm^{-1}]	ϱ$(C\text{-}H)_{as}$ [cm^{-1}]	ϱ$(C\text{-}H)_s$ [cm^{-1}]	SiO_2 nanoparticles + *v*(C-O) [cm^{-1}]	ν(Si-O-Si) [cm^{-1}]	*v*(C-C) [cm^{-1}]	Si-C [cm^{-1}]	ϱ(C-C) [cm^{-1}]
pure PDMS	/	2962	2904	/	1412	1258	/	1054; 1010	/	788	/
LIG/PDMS/PEG10%	3474	2923	2852	1652	/	1384	1097	/	/	807	/
LIG/PDMS/PEG20%	3433	2964	/	1631	/	1384	1093	/	/	805	468
LIG/PDMS/PEG30%	3352	2963	2885	1629	/	/	1071	/	/	800	463
LIG/PDMS/PEG40%	3415	2964	/	1618	1445	/	1093	/	875	807	467

Table S9. Assignation of bands in FTIR spectrum of PDMS/30%PEG composite.

Polymer	ν (O-H) [cm^{-1}]	ν (C-H) [cm^{-1}]	ν (C=O) [cm^{-1}]	ν $(C\text{-}O)_{COOH}$ [cm^{-1}]	ν (Si-O-Si) + ν (C-O-C) [cm^{-1}]	ν (Si-O) [cm^{-1}]	ϱ $(Si\text{-}CH_3)$ [cm^{-1}]
PDMS/30%PEG	3595	2968	1732	1415	1143; 1273	1041	875; 829

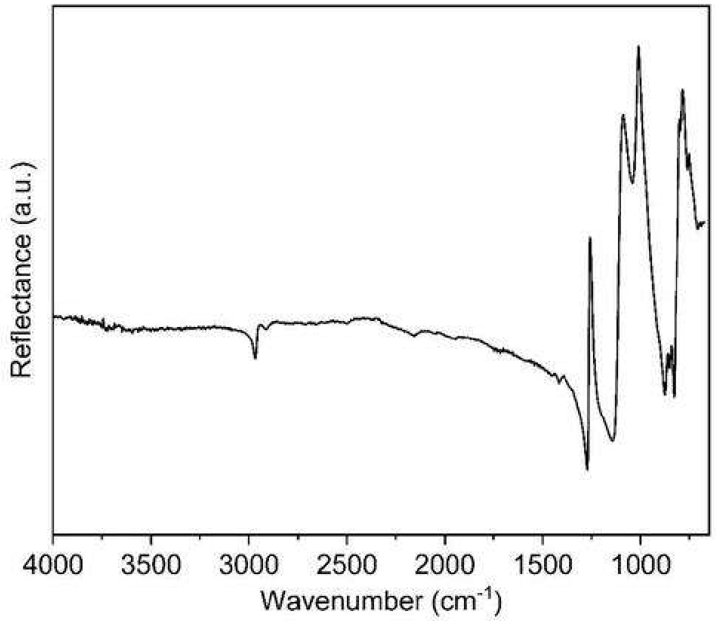

Figure S7. FTIR spectrum of PDMS/30%PEG composite.

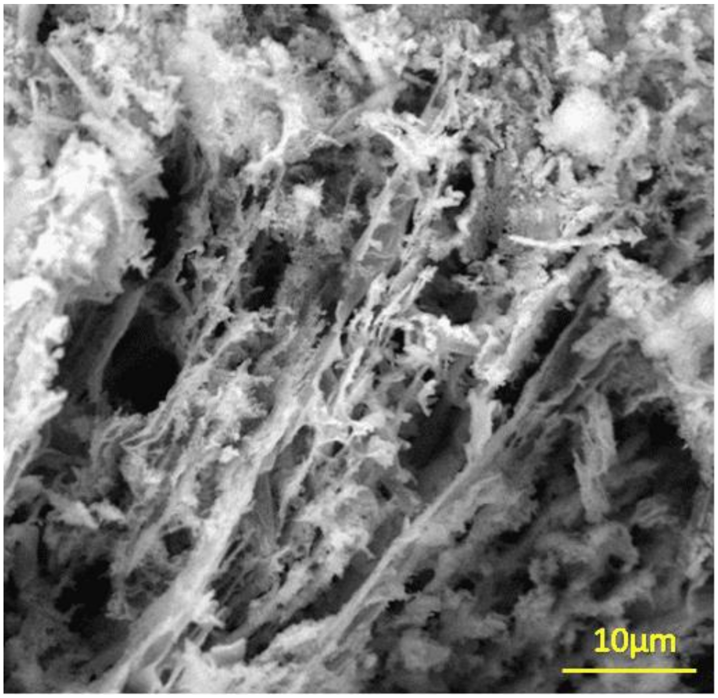

Figure S8. Scanning electron micrograph cross-section of LIG on PDMS/40%PEG.

Table S10. Pore sizes for LIG on PEG with various PEG contents

PEG content (wt. %)	Pore size (μm)
10	3.04
20	0.86
30	1.02
40	0.88

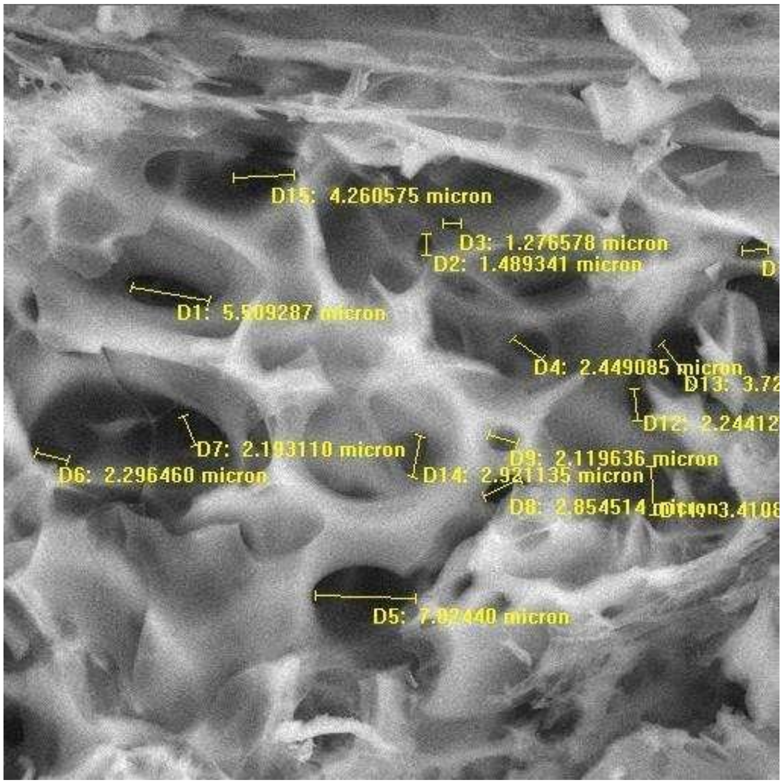

Figure S9. Diameter of pores for LIG/PDMS/10%PEG measured in Image-Pro Plus

SEM-EDX

Analysis of LIG on PDMS/40%PEG made with 45 mm s^{-1} scanning speed and 9 W laser power revealed higher carbon wt.% and lower silicon wt.%, showing that those laser parameters result in the best conversion of PDMS/PEG to graphene. Furthermore, the carbon atomic concentration is greater, while the silicon and oxygen atomic concentrations are lower, for LIG on PDMS/40%PEG produced with a scanning speed of 45 mm s^{-1} and a laser power of 10.2 W, compared to 9.6 W laser power. When comparing samples generated at various scanning speeds, we can see that the material made with 45 mm s^{-1} has the greatest carbon content and the lowest oxygen content compared to the other materials.

Table S11. EDX analysis of LIG on PDMS/40%PEG made with different laser parameters and LIG on PDMS with different PEG content made with the same laser parameters.

	LIG on PDMS/PEG made with 45 mm s^{-1} scanning speed and 9 W laser power				LIG on PDMS/40%PEG made with 9 W laser power			LIG on PDMS/40%PEG made with 45 mm s^{-1} scanning speed	
Atomic concentration (%)	10% PEG	20% PEG	30% PEG	40% PEG	55 mm s^{-1}	45 mm s^{-1}	35 mm s^{-1}	10.2 W	9.6 W
Carbon, C	29.11	23.05	22.77	29.25	22.36	29.25	17.87	27.23	22.29
Oxygen, O	50.33	56.27	52.72	51.73	54.33	51.73	59.09	50.53	54.37
Silicon, Si	20.56	20.68	24.52	19.03	23.32	24.52	23.04	22.24	23.34

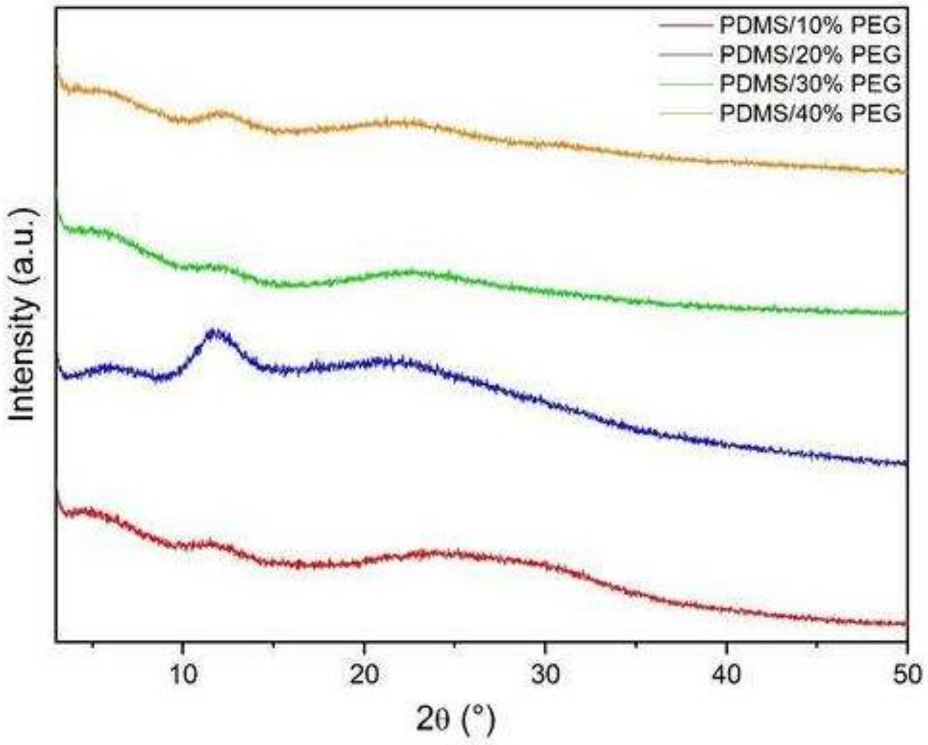

Figure S10. Diffractogram of PDMS/PEG composites with different PEG content.

Table S12. Characteristic diffraction peaks for composite PDMS/PEG, LIG films on PDMS/PEG, and scraped LIG on PDMS/PEG with different PEG content.

Materials	(°)	10% PEG	20% PEG	30% PEG	40% PEG
PDMS/PEG	2θ	11.48	11.96	11.5	12.08
		23.98	22.14	22.62	22.24
LIG films on PDMS/PEG	2θ	35.52	35.5	35.74	35.8
		22.16	21.92	22.28	21.54
		11.86	11.6	11.74	12.1
		41.3	41.24	41.6	41.3
LIG scraped from PDMS/PEG	2θ	23.2	22.76	22.58	22.58
		35.4	35.38	35.4	35.42
		41.16	41.2	41.14	41.18

Table S13. Influence of humidity on LIG/PDMS/PEG

**Sample**	**Electrical resistance for different humidity at 25 °C**			
	40%	60%	80%	90%
**LIG/PDMS/20%PEG**	16.90 kΩ	16.05 kΩ	15.84 kΩ	16.22 kΩ
**LIG/PDMS/30%PEG**	13.78 kΩ	13.10 kΩ	12.46 kΩ	13.70 kΩ
**LIG/PDMS40%PEG**	3.50 kΩ	3.28 kΩ	3.45 kΩ	3.55 kΩ

Table S14. Influence of temperature on LIG/PDMS/PEG

**Sample**	**Electrical resistance for different temperature at 40% humidity**			
	20 °C	30 °C	40 °C	50 °C
**LIG/PDMS/20%PEG**	4.65 kΩ	7.38 kΩ	4.40 kΩ	7.70 kΩ
**LIG/PDMS/30%PEG**	12.78 kΩ	14.28 kΩ	14.23 kΩ	14.70 kΩ
**LIG/PDMS40%PEG**	2.93 kΩ	3.51 kΩ	4.46 kΩ	5.40 kΩ

Table S15. Influence of humidity (90% at 25 °C) on the mechanical properties of LIG/PDMS/PEG

**Sample**	**Young modulus (MPa)**	**Tensile strength (MPa)**	**Elongation at break (%)**
**LIG/PDMS/20%PEG**	0.19	0.12	123.2
**LIG/PDMS/30%PEG**	0.34	0.21	104.0
**LIG/PDMS/40%PEG**	0.43	0.27	86.9

Table S16. Influence of temperature (50 °C at 40% humidity) on the mechanical properties of LIG/PDMS/PEG

**Sample**	**Young modulus (MPa)**	**Tensile strength (MPa)**	**Elongation at break (%)**

**LIG/PDMS/20%PEG**	0.24	0.36	208.40
**LIG/PDMS/30%PEG**	0.17	0.21	215.39
**LIG/PDMS/40%PEG**	0.27	0.28	138.35

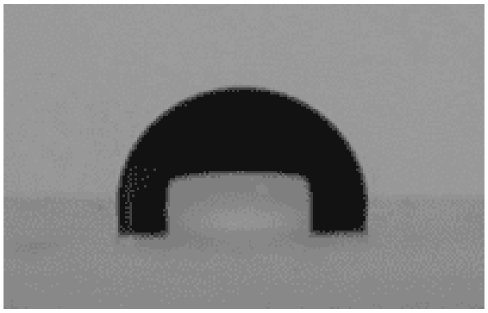

Figure S11. Photograph of a water drop on pure PDMS.

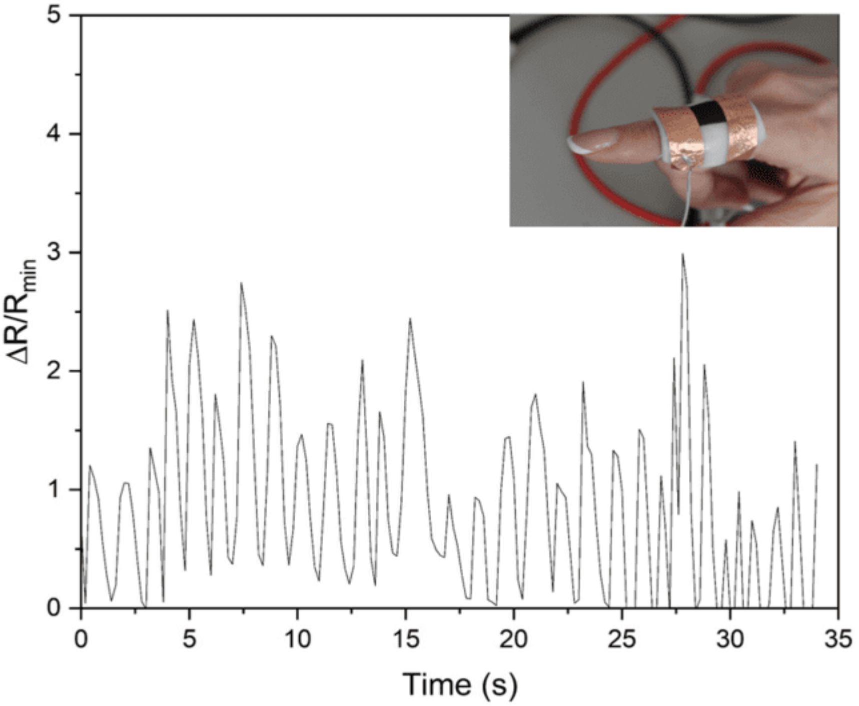

Figure S12. Relative resistance as a function of time in 2 minutes and a picture of a tactile sensor on a thicker substrate (inset)

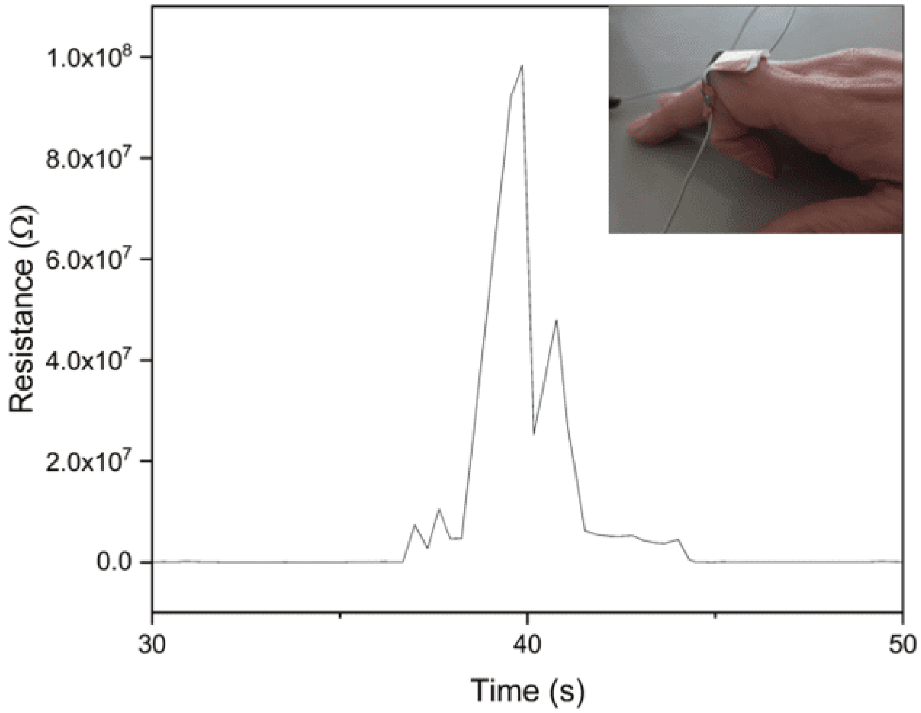

Figure S13. Resistance change with time when the finger is fully bent (90°) and a photograph (inset). The resistance upon bending rises to extremely high values, which makes resistance at rest appear near null on the shown scale.

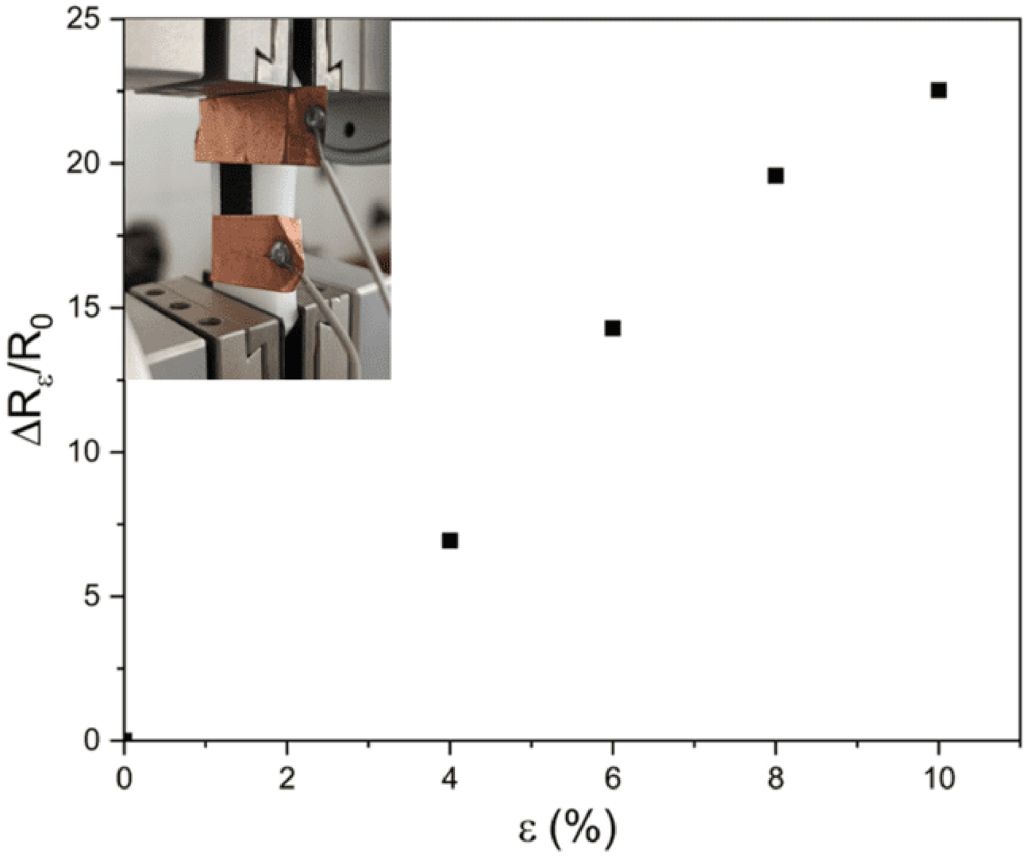

Figure S14. Relative resistance change with applied strain on the sensor and the photograph of the sensor before applying strain (inset)

Table S17. Measured parameters for the calculation of the Gauge factor

ε (%)	R_0 (Ω)	R_ε (Ω)	L_0 (cm)	L_ε (cm)	GF
**2**	3.4	22	2	2.026	420.75
**4**	3.4	27	2	2.052	266.91
**6**	3.4	52	2	2.078	366.51
**8**	3.4	70	2	2.104	376.69
**10**	3.4	80	2	2.130	346.88

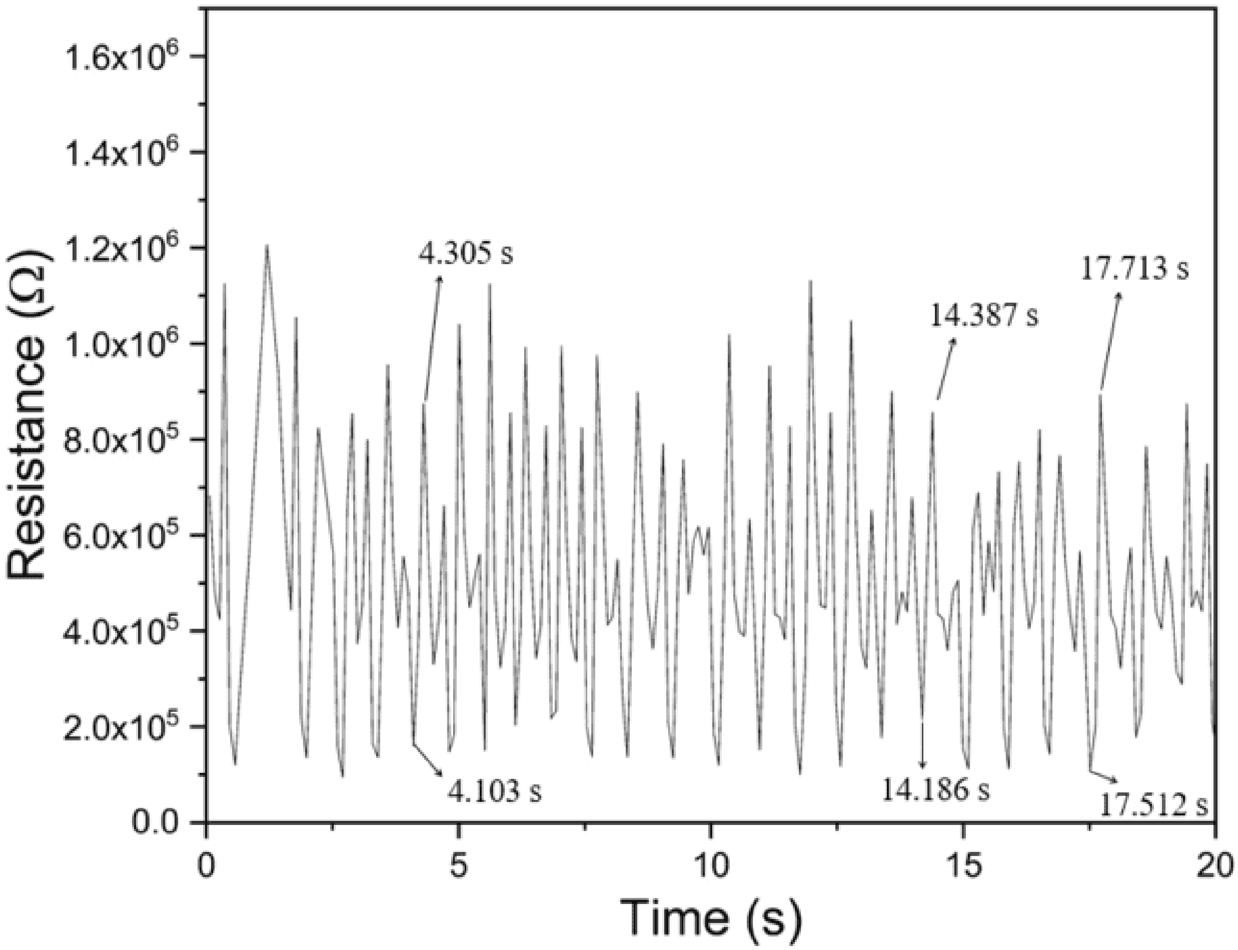

Figure S15. Measurements of the response time of the tactile sensor

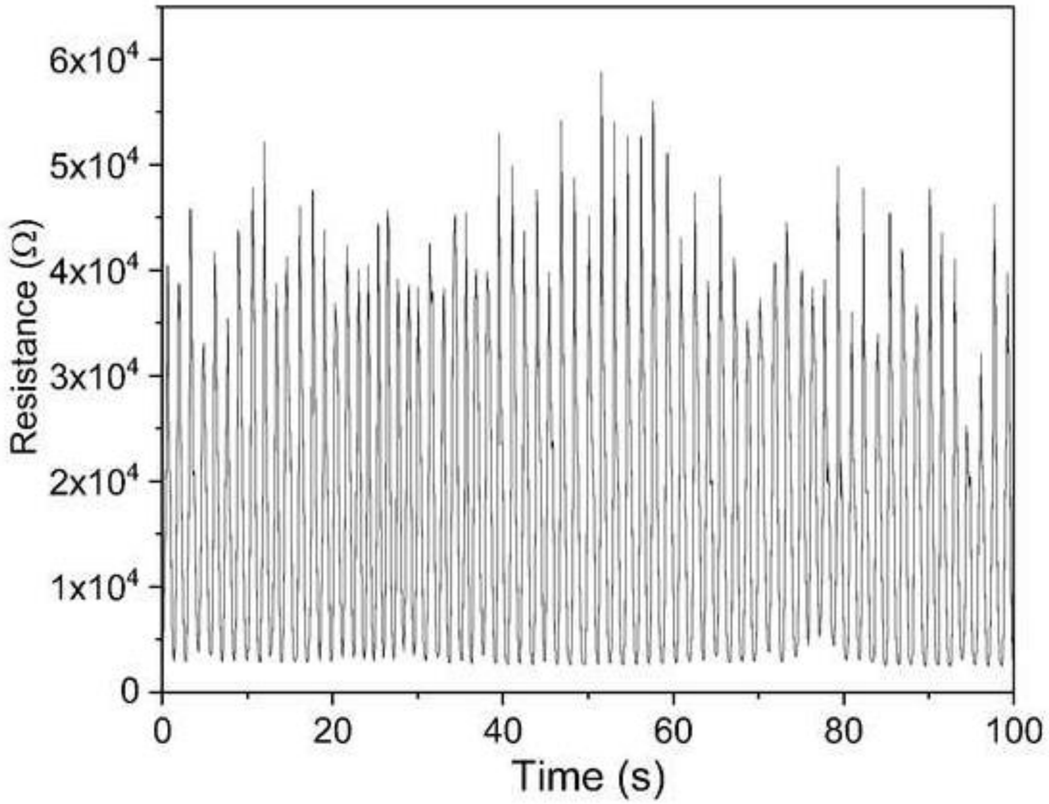

Figure S16. Repeated bending of the finger at an angle of ~50°

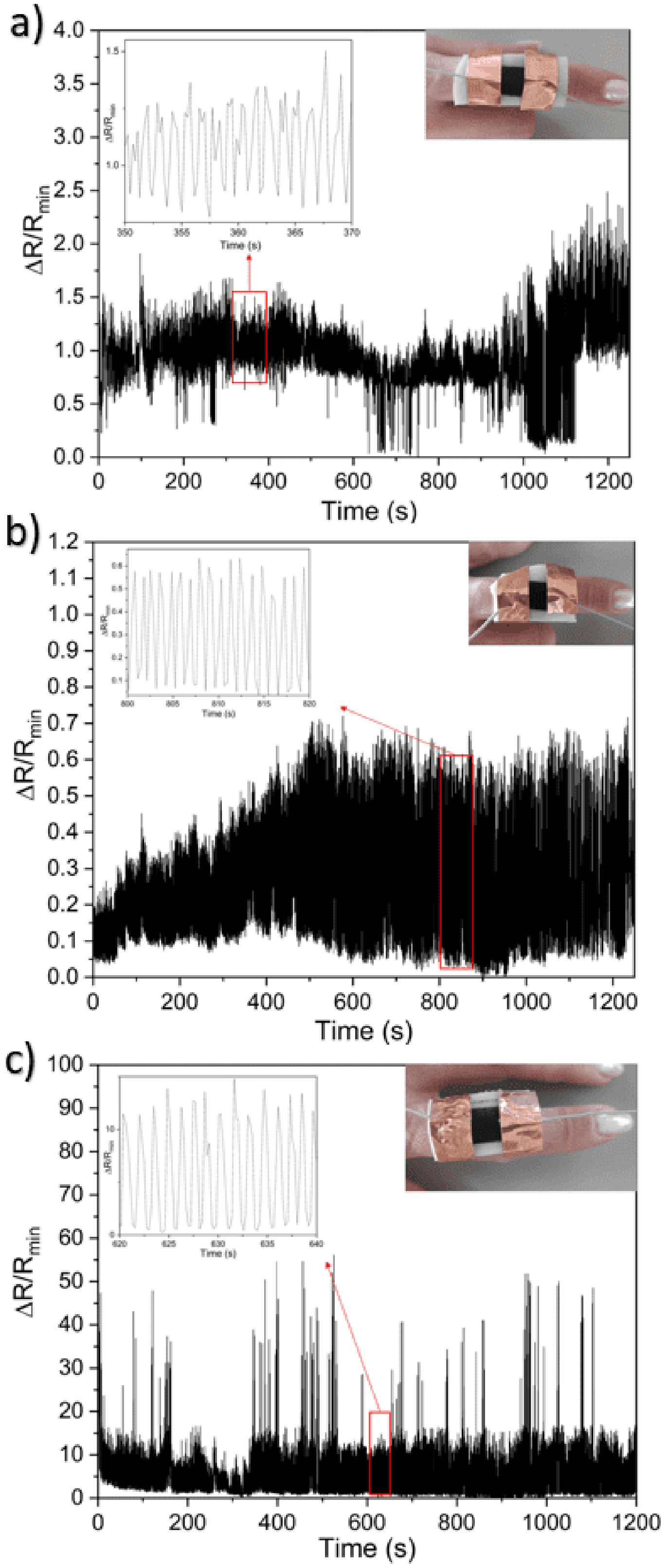

a)
ΔR/R_min
Time (s)
0.0
0.5
1.0
1.5
2.0
2.5
3.0
3.5
4.0
0
200
400
600
800
1000
1200
b)
ΔR/R_min
Time (s)
0.0
0.1
0.2
0.3
0.4
0.5
0.6
0.7
0.8
0.9
1.0
1.1
1.2
c)
ΔR/R_min
Time (s)
0
10
20
30
40
50
60
70
80
90
100

Figure S17. Sensing response to finger bending across 1000 cycles (20 minutes) with LIG-based sensors on (a) PDMS/20%PEG, (b) PDMS/30%PEG, and c) PDMS/40%PEG, with inset photographs of the sensors. The finger was bent at an angle of 90°.

Table S18. Gauge factor of LIG/PDMS-based sensors with different PEG content

Materials	Gauge factor; strain range
LIG on PDMS/20%PEG	18.46; ε = 0-10%
LIG on PDMS/30%PEG	12.73; ε = 0-10%
LIG on PDMS/40%PEG	190.45; ε = 0-10%

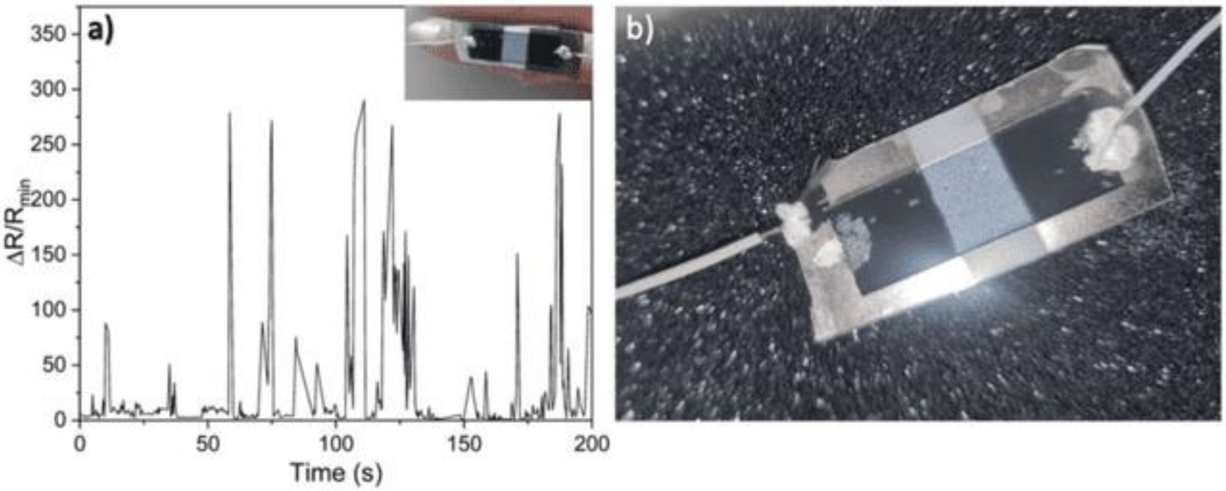

Figure S18. a) Finger bending measurements with directly induced graphene on PDMS/40%PEG. Inset and b) depict photographs of the working sensor and sensor with detached contacts.
